# A tRNA fragment, tRF5-Glu, regulates BCAR3 expression and proliferation in ovarian cancer cells

**DOI:** 10.18632/oncotarget.20709

**Published:** 2017-09-08

**Authors:** Kun Zhou, Kevin W. Diebel, Jon Holy, Andrew Skildum, Evan Odean, Douglas A. Hicks, Brent Schotl, Juan E. Abrahante, Monique A. Spillman, Lynne T. Bemis

**Affiliations:** ^1^ Department of Biomedical Sciences, University of Minnesota, Duluth, MN, 55812, USA; ^2^ Division of Reproductive Sciences, Department of Obstetrics and Gynecology, University of Colorado Denver, Anschutz Medical Campus, Aurora, CO, 80045, USA; ^3^ University of Minnesota Informatics Institute, University of Minnesota, Minneapolis, MN, 55455, USA; ^4^ Texas A&M University Medical School, Baylor University Medical Center, Dallas, TX, 75206 USA

**Keywords:** tRNA fragments, BCAR3, ovarian cancer, tRF5-Glu, noncoding RNA

## Abstract

Ovarian cancer is a complex disease marked by tumor heterogeneity, which contributes to difficulties in diagnosis and treatment. New molecular targets and better molecular profiles defining subsets of patients are needed. tRNA fragments (tRFs) offer a recently identified group of noncoding RNAs that are often as abundant as microRNAs in cancer cells. Initially their presence in deep sequencing data sets was attributed to the breakdown of mature tRNAs, however, it is now clear that they are actively generated and function in multiple regulatory events. One such tRF, a 5’ fragment of tRNA-Glu-CTC (tRF5-Glu), is processed from the mature tRNA-Glu and is shown in this study to be expressed in ovarian cancer cells. We confirmed that tRF5-Glu binds directly to a site in the 3’UTR of the Breast Cancer Anti-Estrogen Resistance 3 (BCAR3) mRNA thereby down regulating its expression. BCAR3 has not previously been studied in ovarian cancer cells and our studies demonstrate that inhibiting BCAR3 expression suppresses ovarian cancer cell proliferation. Furthermore, mimics of tRF5-Glu were found to inhibit proliferation of ovarian cancer cells. In summary, BCAR3 and tRF5-Glu contribute to the complex tumor heterogeneity of ovarian cancer cells and may provide new targets for therapeutic intervention.

## INTRODUCTION

Worldwide, ovarian cancer is a leading cause of cancer mortality among women, with the five-year survival rate for women with advanced stage disease expected to be less than 30% [1]. The poor survival is attributed to several factors including nonspecific symptoms of early disease, late stage of diagnosis, and the molecular heterogeneity of the disease [1, 2]. A recent summary and review of microarray studies of tissue samples from ovarian cancer patients demonstrates that heterogeneity is associated with the wide diversity of patient outcomes [3]. In addition to the molecular heterogeneity of mRNA expression, it is now being realized that regulation by noncoding RNAs contributes to the diversity of gene expression in cancer patients [4, 5].

The impact of newly identified regulatory mechanisms of small noncoding RNAs on the heterogeneity of gene expression in cancer is exemplified by the recent explosion of microRNA studies including their expression in ovarian cancer [6–9]. Furthermore, due to an increase in studies utilizing high throughput sequencing technologies even more prevalent classes of noncoding RNA are being identified [10, 11]. One group of recently identified noncoding RNAs implicated in cancer biology are the tRNA fragments (tRFs) [12–14]. tRFs are evolutionarily conserved, from prokaryotes to eukaryotes, and include segments of tRNAs from both pre-tRNAs and mature tRNAs [11]. Due to their recent identification, the naming convention for members of the tRFs is not yet consistent [13, 15, 16]. The tRFs as a class include but are not limited to tRNA halves, tiRNAs, tsRNAs, intermediate tRFs, 5’tRFs, 3’tRFs, and SHOT-RNAs [17–21].

The biogenesis of tRFs is an active process resulting from the cleavage of tRNAs by multiple enzymes including Angiogenin (ANG), tRNAse Z, and Dicer [17–19]. The potential functions of tRFs are the focus of many current studies. tRFs have been isolated as part of Argonaute (AGO) complexes implying that one function of a subset of tRFs may be similar to that of microRNAs [12, 22, 23]. For instance studies initially aimed at identifying microRNA targets, including HTS-CLIP, PAR-CLIP and CLASH studies, have been reanalyzed to confirm the association of tRFs with mRNAs from numerous cellular sources, including a subset from cancer cell lines [12, 15, 22, 24]. An association with AGO implies that tRFs may be regulators of mRNA expression. To date, however, only a few direct mRNA targets of tRFs have been confirmed [22, 25].

A subset of tRFs were initially misclassified as microRNAs and were eventually discovered and eliminated from miRBase if their genomic location was near or embedded in a tRNA gene [26, 27]. For example, miR-2476, which is one base pair different from the 5’-tRF derived from tRNA-Glu-CTC (tRF5-Glu), was discovered in the cow and pig and is now called a dead entry because of its location near a known tRNA (miRBase Accession: MI0011537) [28]. Prior to its elimination from miRbase, miR-2476 was included in TargetScan (6.2) for the cow and predicted to bind the 3’ untranslated region (UTR) of many potential mRNA targets. In a pilot study, we found that tRF5-Glu is present in the urine of ovarian cancer patients (publication in press) and questioned if it might be present in ovarian cancer cells.

In order to determine if tRF5-Glu is expressed and functional in ovarian cancer cells, we examined its expression in five ovarian cancer cell lines. We have shown that tRF5-Glu is present in ovarian cancer cells grown in culture and is capable of directly binding a predicted target, the Breast Cancer Anti-Estrogen Resistance gene 3 (BCAR3). BCAR3 has been well studied in breast cancer for its role in anti-estrogen resistance and breast cancer cell proliferation [29]. BCAR3 is also known to be expressed in B-cells, colorectal cancer, and more recently has been shown to function during development [30–33]. However, regulatory mechanisms governing the expression of BCAR3 remain poorly understood. The aim of this study was to identify the expression pattern, function and regulation of BCAR3 and tRF5-Glu in ovarian cancer cells. In this study, we show decreasing BCAR3 expression and increasing tRF5-Glu inhibits the proliferation of ovarian cancer cells. These studies begin to define the regulatory mechanisms of tRFs in ovarian cancer, providing a potential new class of molecules to target for therapeutic development.

## RESULTS

### tRF5-Glu is expressed in ovarian cancer cell lines

**Figure 1:**
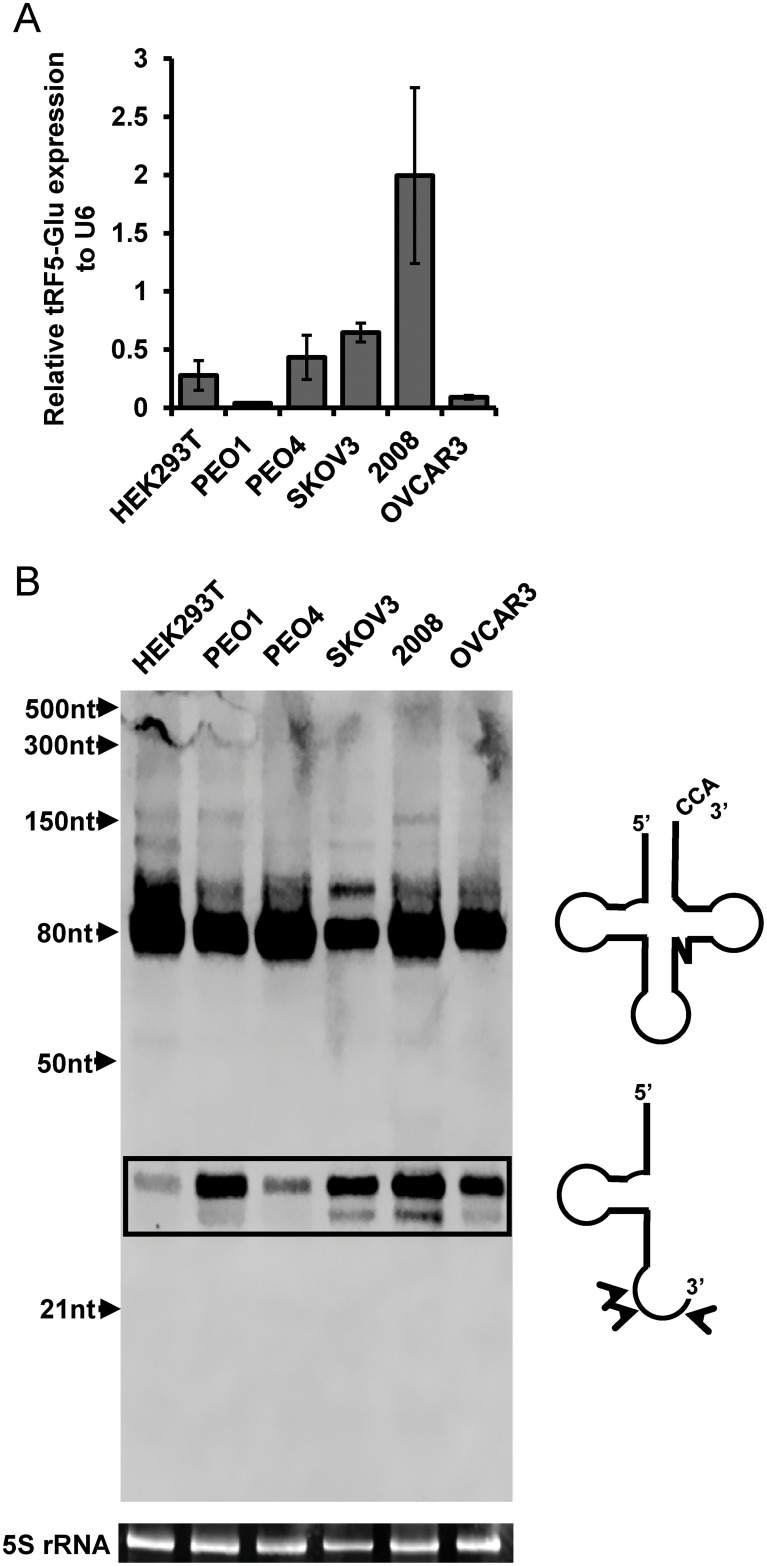
tRF5-Glu is expressed in ovarian cancer cell lines and HEK293T cells Ovarian cancer cells were grown to confluence and lysed in passive lysis buffer for fifteen minutes prior to RNA extraction. **(A)** tRF5-Glu expression was assessed using qRT-PCR. tRF5-Glu expression is shown relative to the expression of the internal control, RNU6B (U6). qRT-PCR experiments are replicates of three independent experiments and error bars refer to +/- standard error. **(B)** Validation of tRF5-Glu expression by Northern blot analysis. Northern analysis utilized a 5’ biotinylated probe designed to hybridize antisense to the tRF5-Glu region of tRNA-Glu. This probe binds unprocessed tRNA-Glu (greater than 80 base pairs), mature tRNA-Glu (70-80 base pairs) and tRF5-Glu (29-35 base pairs, box). A schematic of the mature tRNA-Glu and the tRF5-Glu is included next to the northern blot showing the mature tRNA-Glu (top) and predominant cleavage sites for tRF5-Glu (bottom). Potential cleavage sites resulting in various size fragments are shown with arrowheads on the schematic of tRF5-Glu. Ethidium bromide stained 5S rRNA below the blot serves as a loading control for the Northern analysis.

In order to confirm that ovarian cancer cells express tRF5-Glu, we interrogated a panel of ovarian cancer cell lines by quantitative real-time PCR (qRT-PCR) and Northern analysis. The five ovarian cancer cell lines included PEO1, PEO4, SKOV3, 2008, OVCAR3 and were chosen for their molecular heterogeneity and previous use in studies of ovarian cancer [34–36]. The cell line HEK293T was used as a positive control because the expression of tRF5-Glu in this cell line had been previously described in RNA sequencing studies (RNA-seq) [23]. Expression of tRF5-Glu was measured by qRT-PCR in these cell lines (Figure 1A). Northern Blot analysis provided further confirmation that tRF5-Glu is expressed in ovarian cancer cells (Figure 1B).

The ovarian cancer cell lines and HEK293T expressed tRNA-Glu and tRF-Glu fragments. The full length mature tRNA-Glu is visible as larger than 70 nucleotides as expected. The tRF5-Glu variants range in size from 29-35 nucleotides (Figure 1B, box). Models of the mature tRNA and the tRFs are included next to the corresponding bands of the Northern analysis (Figure 1B). The Northern analysis revealed that the ovarian cancer cell lines were able to express tRF5-Glu.

### ANG modulates the biogenesis of tRF5-Glu in ovarian cancer cells

ANG is an RNase implicated in the biogenesis of the class of tRFs known as the tRNA-halves [37] and the tiRNAs [17]. The tRFs observed by northern blot correspond to the size expected for tRNA-halves. We performed RNAi knockdown of ANG expression in ovarian cancer cell lines to determine if ANG has a role in generating tRF5-Glu in ovarian cancer cells. Two ANG siRNAs were shown to significantly reduce ANG protein expression as compared with a control siRNA in the cell lines (Supplementary Figure 1).

**Figure 2:**
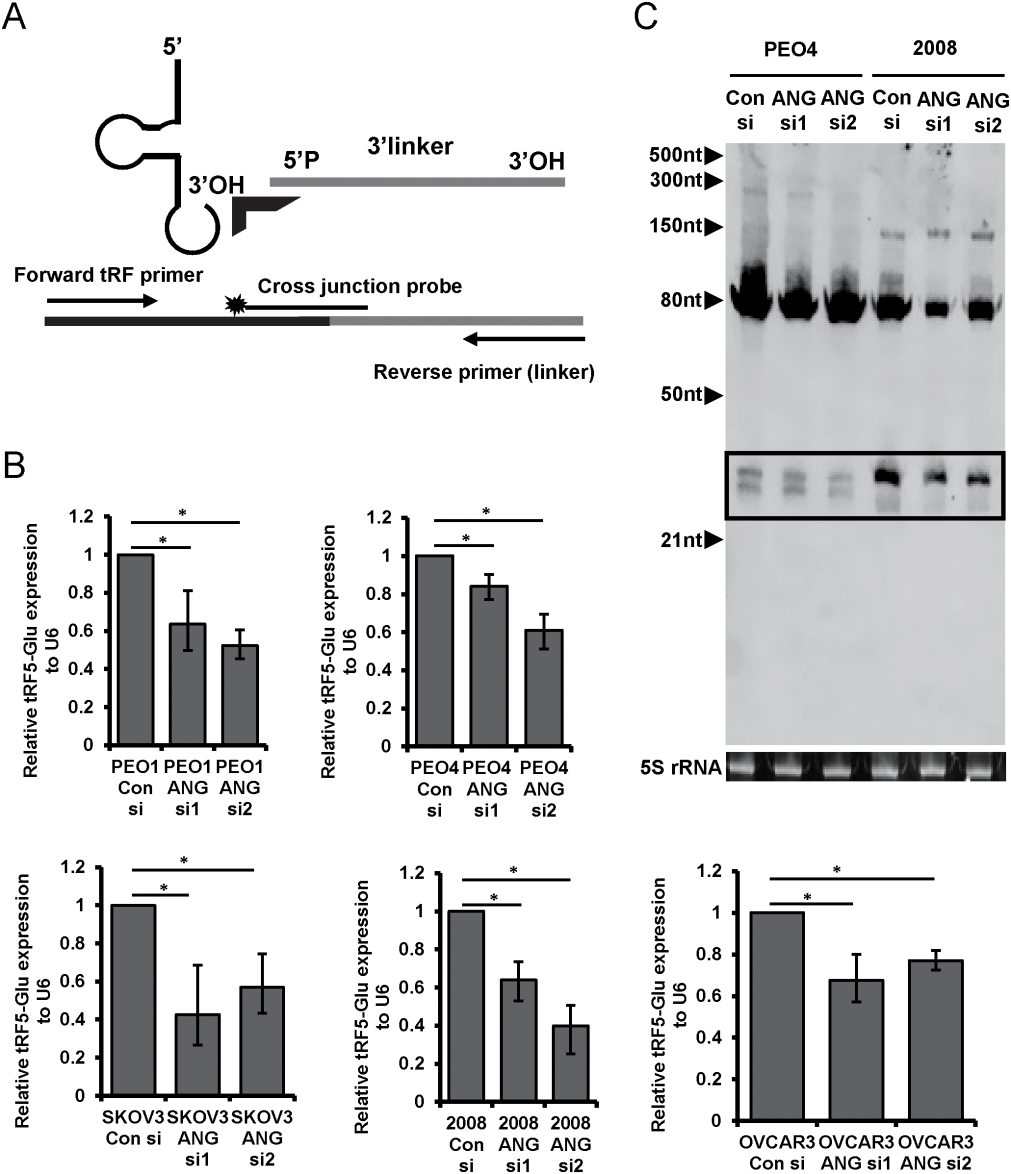
The biogenesis of tRF5-Glu is regulated by ANG in ovarian cancer cells ANG expression was blocked by transfection of cells with ANG siRNA to determine if tRF5-Glu expression is altered. **(A)** Ligation PCR provides a method to quantify tRF5-Glu, a diagram of the primer placement for Ligation PCR is included. A labeled probe covering the junction between the end of the 3’ linker and target sequence is shown. The linker is designated in grey, the cross junction probe has a star and arrows designate the primers. **(B)** Ligation PCR of cDNA from PEO1, PEO4, SKOV3, 2008, and OVCAR3 cells transfected with control and ANG siRNAs was conducted. tRF5-Glu expression is compared relative to the internal control U6. Ligation PCR experiments are replicates of three independent experiments and error bars refer to +/- standard error (^*^ p-value <.05). **(C)** Northern analysis of RNA collected from cells transfected with a control siRNA and two ANG specific siRNAs (ANG siRNA1 and ANG siRNA2). The tRF5-Glu bands are shown within a box.

tRF5-Glu is part of the mature tRNA-Glu and it is not possible to accurately measure the fragment by routine qRT-PCR without also amplifying the mature tRNA. In order to accurately quantify tRF5-Glu using qRT-PCR methods, we modified an assay previously described by Honda et al., [21] (Figure 2A). The modified method is called ligation PCR and includes an internal control of U6 and a specific primer for tRF5-Glu (described in methods and materials). Ligation PCR confirmed that the tRNA half, tRF5-Glu, is reduced in all five cell lines treated with ANG siRNA (Figure 2B). We also confirmed changes in tRF5-Glu by Northern analysis in two cell lines treated with siRNAs to ANG (Figure 2C). The loading control of 5S rRNA was used to show equal loading and we did observe a reduction in the mature tRNA-Glu, as has been shown in a recent study of another tRF [38]. Taken together ligation PCR and Northern analysis confirm that ANG participates in the biogenesis of tRF5-Glu in ovarian cancer cells.

### tRF5-Glu and its predicted target BCAR3 are simultaneously expressed in ovarian cancer cells

A variant of tRF5-Glu was previously misreported to be microRNA-2476 (miRBase accession number: MI0011537). microRNA-2476, now recognized as a variant of tRF5-Glu, was included in TargetScan version 6.2 and thus a list of potential mRNA targets for miR-2476 is available. A somewhat controversial question about tRFs is if they function in a manner similar to microRNAs. We examined the list of predicted targets of miR-2476 in TargetScan 6.2 (available at http://www.targetscan.org/vert_61/) and selected potential targets for further analysis based on their previous expression in cancer (Supplementary Table 1).

BCAR3 was selected from the genes predicted for regulation due to its expression in the ovarian cancer intraperitoneal xenograft model previously published [34] and available from GEO Profiles (GDS4066:204032). The available GEO data indicates that both PEO4 and 2008 cells express the mRNA of BCAR3. In order for BCAR3 mRNA to be targeted by tRF5-Glu, they both must be present in cells grown under the same conditions. To determine if BCAR3 and tRF5-Glu are present in ovarian cancer cells, PEO4 and 2008 cells were grown in the presence or absence of estrogen for 48 hours, similar to methods used in the GEO experiment [34].

**Figure 3:**
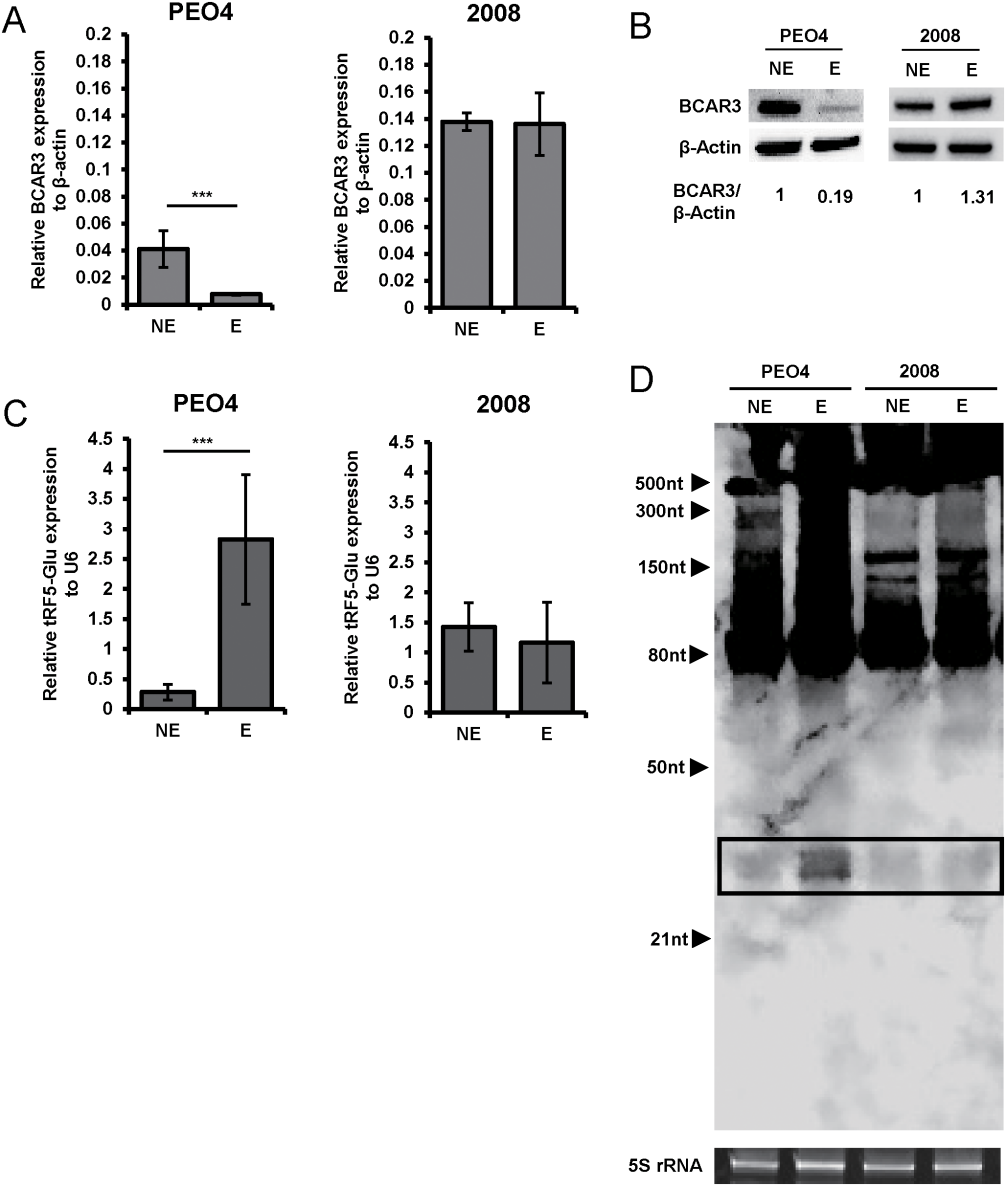
tRF5-Glu and its potential target BCAR3 are expressed in ovarian cancer cell lines Ovarian cancer cell lines were grown under estrogen starved (NE) or estrogen stimulated (E) conditions to determine the best conditions to study the potential relationship between tRF5-Glu and BCAR3. **(A)** Expression of BCAR3 mRNA in cDNA from PEO4 and 2008 cells estrogen starved (NE) or estrogen stimulated (E) as assayed by qRT-PCR. qRT-PCR experiments are replicates of three independent experiments and error bars refer to +/- standard error (^**^ p-value <.01 and ^***^ p-value<.005). **(B)** Western analysis of BCAR3 protein expression at 48 hours of estrogen starvation (NE) or estrogen stimulation (E) in the two ovarian cancer cell lines. Densitometry of each band was measured and the relative ratio of BCAR3/β-Actin is shown. **(C)** Expression of tRF5-Glu amplified from cDNA of PEO4 and 2008 cells estrogen starved (NE) or estrogen stimulated (E) as assayed by ligation PCR. **(D)** Northern analysis of tRF5-Glu in estrogen starved (NE) or estrogen stimulated (E) PEO4 and 2008 cells. Cells were grown for 48 hours in estrogen depleted media and then the media was replaced by new estrogen depleted media (NE) or media supplemented with estrogen for an additional 48 hours (E). Cells were then lysed by direct addition of Qiazol for RNA extraction. The tRF5-Glu bands are shown within a box.

PEO4 and 2008 cells were both shown to express BCAR3 as well as tRF5-Glu under both growth conditions. Interestingly, BCAR3 expression decreased in estrogen stimulated PEO4 cells compared to estrogen starved PEO4 cells. However, there was no significant difference in BCAR3 expression between estrogen treated or estrogen deprived 2008 cells (Figure 3A and 3B). In contrast, tRF5-Glu expression was increased in the PEO4 cell line stimulated with estrogen, while again no change was observed in the 2008 cell line (Figure 3C and 3D, box). These data demonstrate a potential correlation between BCAR3 and tRF5-Glu under estrogen stimulated growth conditions in PEO4, while no significant change in BCAR3 or tRF5-Glu was observed in 2008 cells.

### tRF5-Glu directly binds BCAR3 mRNA in ovarian cancer cells

**Figure 4:**
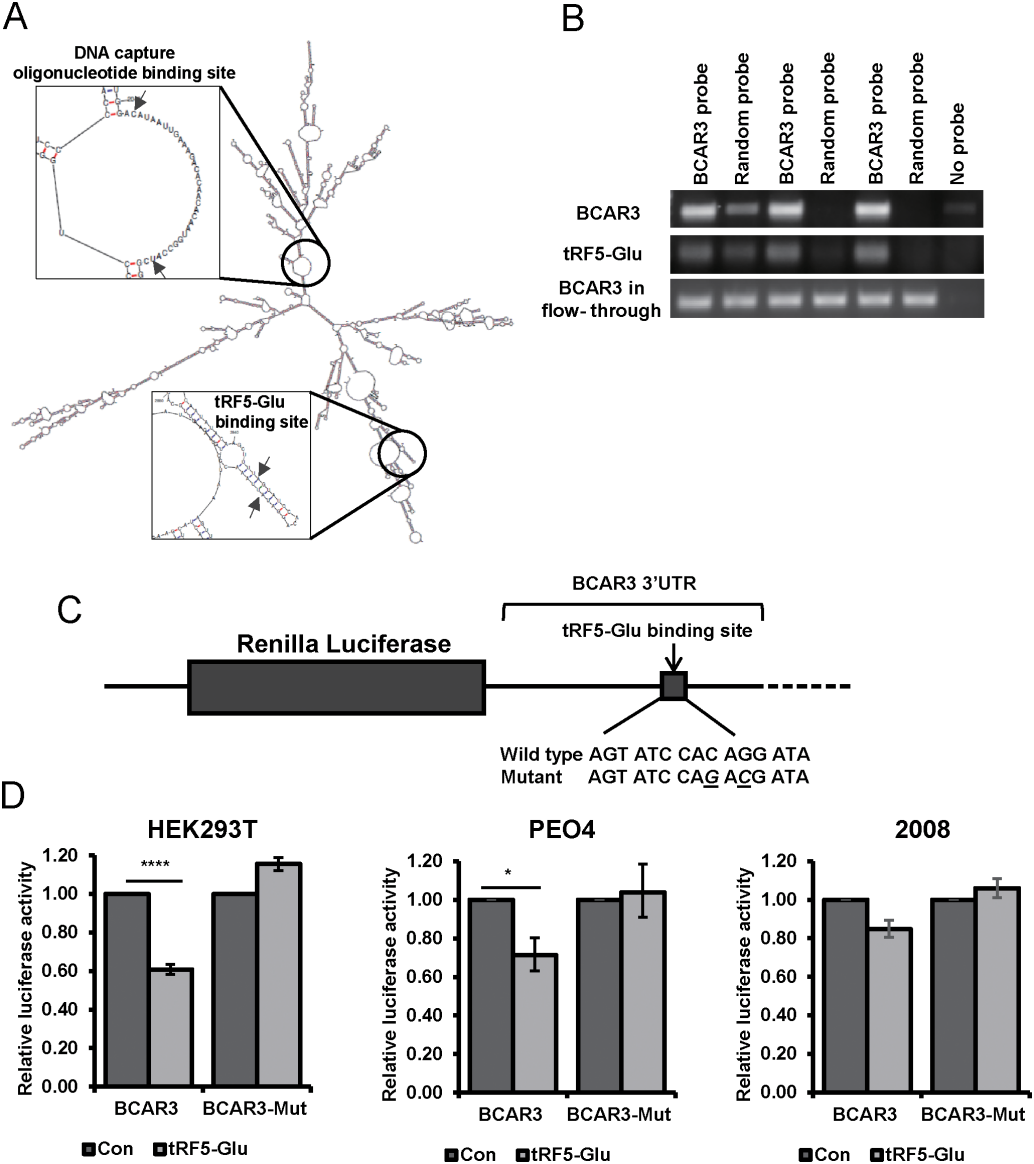
Binding of the BCAR3 mRNA by tRF5-Glu is demonstrated using miR-Catch and luciferase assays **(A)** Secondary structure of the BCAR3 mRNA with placement of the DNA capture probe in relationship to the tRF5-Glu binding site is shown with arrows. **(B)** Triplicate samples of PEO4 cell lysates probed with either BCAR3 or a random probe were amplified by RT-PCR for BCAR3 and tRF5-Glu expression. The BCAR3 mRNA in the flow-through was amplified and is shown for each sample. **(C)** A schematic of the BCAR3 3’UTR and predicted binding site for tRF5-Glu is shown with the wild type and mutant seed sequences listed below. Luciferase reporter plasmids were constructed containing the predicted binding site intact or mutated at two base pairs within the predicted seed region (designated by underlined letters). **(D)** Luciferase constructs with the wild type or mutant BCAR3 3’UTR were transfected into HEK293T, PEO4 and 2008 cells with a control mimic or a mimic of tRF5-Glu. Transfections were performed in triplicate. The result was compared to a control mimic and error bars refer to +/- standard error (^*^ p-value <.05 and ^****^ p-value <.001).

In order to confirm direct binding of the 3’UTR of BCAR3 by tRF5-Glu we modified the previously described miR-Catch assay [39]. We used a biotinylated probe targeting a region of BCAR3 predicted to have an open secondary structure upon RNA folding and distal to the predicted binding site for tRF5-Glu (Figure 4A). Cell lysates from PEO4 cells treated with estrogen for 48 hours were used because we had previously observed regulation of BCAR3 expression in this cell line with these growth conditions (Figure 3). Lysates were collected and hybridized to a BCAR3 specific biotinylated capture probe or a random probe (Figure 4B). BCAR3 mRNA was enriched by pulldown with a BCAR3 probe while it was not enriched by pulldown with a randomized probe. Pulldown with a BCAR3 specific probe showed enrichment for tRF5-Glu.

To investigate if there is direct regulation of BCAR3 by tRF5-Glu, comparable to that expected of a microRNA, a reporter binding assay placing the 3’UTR of BCAR3 downstream of the luciferase reporter was developed. Another reporter construct with two mutations in the predicted binding site was also generated (Figure 4C). HEK293T, PEO4 and 2008 cells were transfected with these constructs in the presence of either a control or a specific mimic of tRF5-Glu. Forty-eight hours after transfection there was a significant 40% and 30% reduction in luciferase activity with the wildtype construct as compared to control in HEK293T and PEO4, respectively. A significant reduction in luciferase activity was not observed in 2008 cells (Figure 4D). There was no significant reduction in luciferase activity with the luciferase assay containing mutations in the predicted miR-2476/tRF5-Glu binding site in HEK293T, PEO4 or 2008 (Figure 4D). These results confirm that BCAR3 is directly regulated by tRF5-Glu. However, the lack of response to tRF5-Glu mimics in 2008 cells suggests an alternative mechanism of BCAR3 regulation in this cell line.

### tRF5-Glu directly regulates BCAR3 expression in ovarian cancer cells

**Figure 5:**
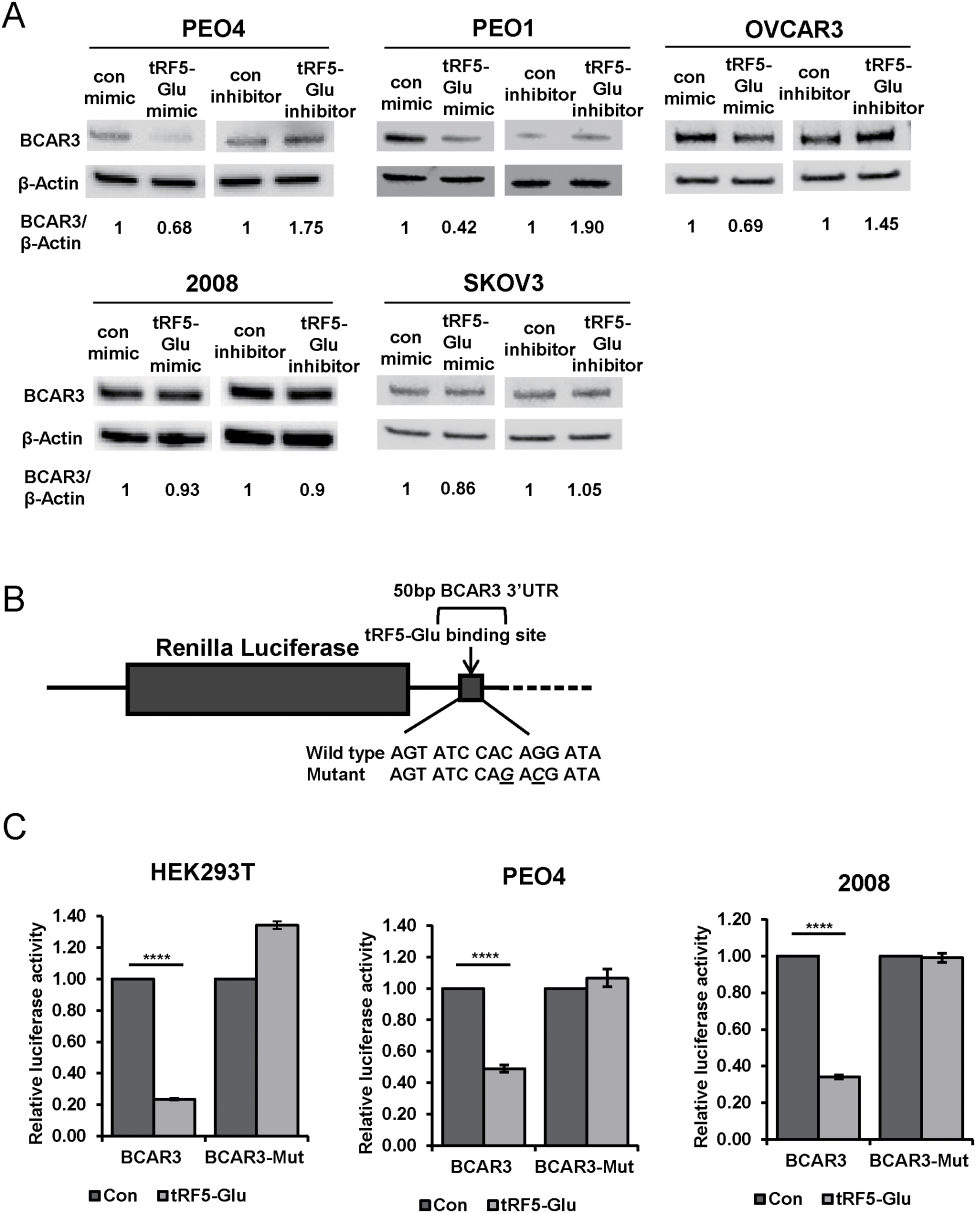
Direct regulation of BCAR3 by tRF5-Glu is predicted in TargetScan 6. 2 which shows a binding site for miR-2476/tRF5-Glu in the BCAR3 3’UTR Mimics and inhibitors of tRF5-Glu were used to determine if BCAR3 is regulated by tRF5-Glu. **(A)** Western analysis was conducted on protein extracts from PEO4, PEO1, OVCAR3, 2008 and SKOV3 cells, estrogen starved and transfected with either a control mimic, a control inhibitor, a tRF5-Glu mimic or a tRF5-Glu inhibitor. Protein lysates were collected at 48 hours post transfection. Densitometry of each band was measured and the relative ratio of BCAR3/β-Actin is shown. **(B)** A schematic of the 50 base pair region of the BCAR3 3’UTR and predicted binding site for tRF5-Glu is shown with the wild type and mutant seed sequences listed below. Luciferase reporter plasmids were constructed containing the predicted binding site intact or mutated at two base pairs within the predicted seed region (designated by underlined letters). **(C)** Luciferase constructs with the wild type or mutant 50bp region of BCAR3 3’UTR were transfected into HEK293T, PEO4 and 2008 cells with a control mimic or a mimic of tRF5-Glu. Transfections were performed in triplicate. The result was compared to a control mimic and error bars refer to +/- standard error (^****^ p-value <.001).

Further investigation of the regulation of BCAR3 by tRF5-Glu was studied in five cell lines, PEO1, PEO4, SKOV3, 2008, and OVCAR3 under growth conditions where BCAR3 was readily expressed. All five ovarian cancer cell lines were transfected with control mimics and inhibitors or a tRF5-Glu specific mimic or inhibitor. Western analysis for BCAR3 protein expression was conducted on cell lysates following 48 hours of treatment (Figure 5A).

In response to treatment with a mimic of tRF5-Glu, BCAR3 expression was observed to decrease in PEO1, PEO4 and OVCAR3 cells as compared to a control mimic; however, in SKOV3 and 2008 cells, BCAR3 protein expression remained unchanged. PEO1, PEO4 and OVCAR3 cells also increased expression of BCAR3 after receiving an inhibitor of tRF5-Glu compared to a control, whereas BCAR3 expression in SKOV3 and 2008 cells was unaltered.

In order to further explore the regulation of BCAR3 by tRF5-Glu we constructed vectors expressing only a 50 base pair region of the 3’ UTR of BCAR3 (Figure 5B). The wild type and mutant constructs were transfected into HEK293T, PEO4 and 2008 cells (Figure 5C). When the smaller region of the BCAR3 UTR is included in the luciferase construct, regulation through the predicted binding site is possible regardless of the cell line where the luciferase constructs are expressed. These results suggest that other regions of the BCAR3 UTR are required for the lack of regulation of BCAR3 by tRF5-Glu observed in 2008 cells.

### Usage of an alternative poly-A site may modulate tRF5-Glu regulation of BCAR3 expression in 2008 ovarian cancer cells

The lack of response of BCAR3 expression to mimics or inhibitors of tRF5-Glu in SKOV3 and 2008 was reminiscent of microRNA studies where the 3’UTR in rapidly growing cells may be shortened thereby deleting a binding site for a microRNA repressor [40–42]. The 3’UTR of mRNAs contain many regulatory sites including, but not limited to, noncoding RNA binding, RNA binding protein binding sites and alternative poly A sites (APA) [43]. It has been shown that APA sites may be used in some rapidly proliferating cells, thereby eliminating a regulatory region and shortening a normally longer 3’UTR [44]. Regulation of mRNA expression through RNA binding sites in the 3’UTR are often affected by the use of tandem APA sites [45].

**Figure 6:**
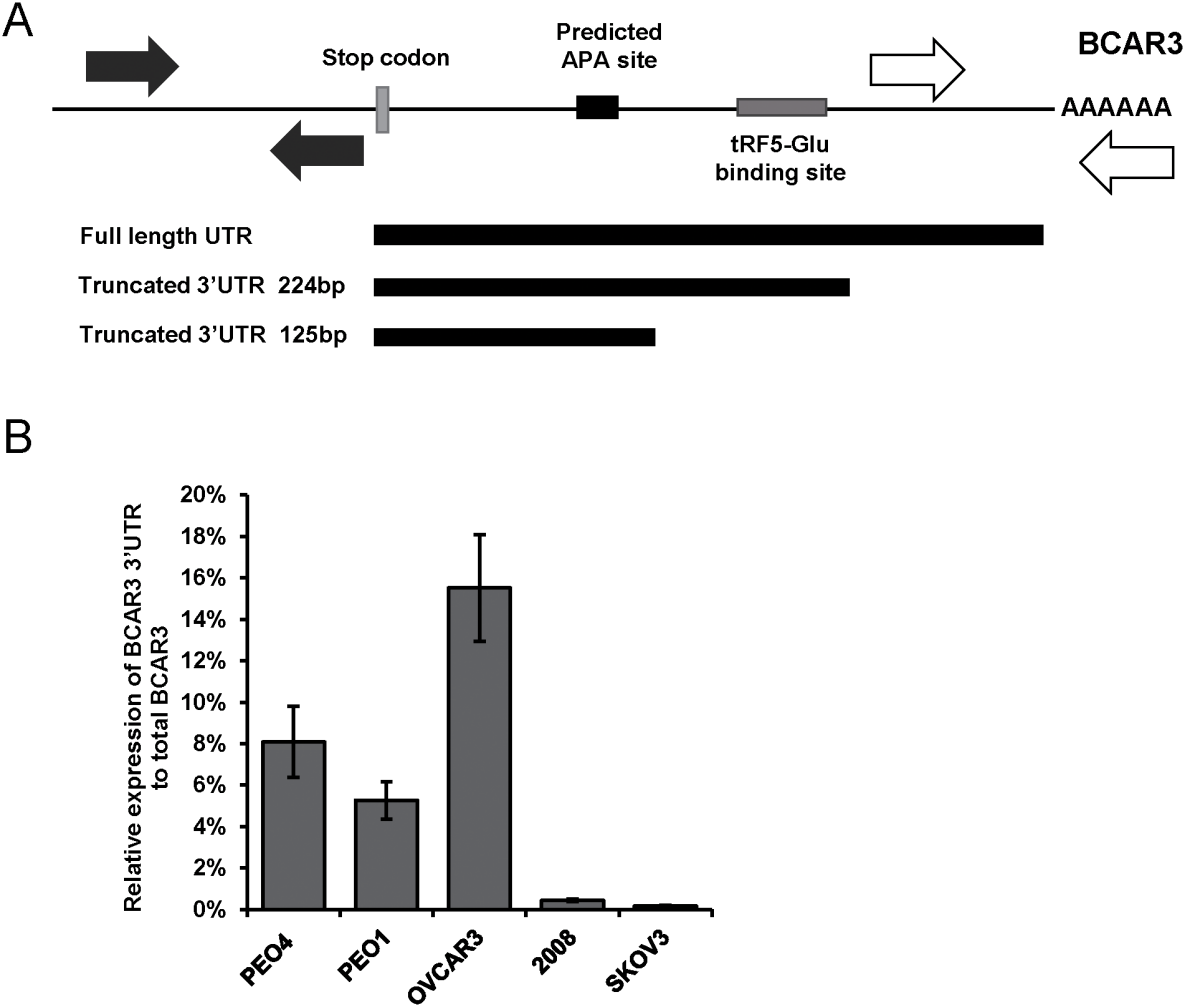
APA sites in the 3’UTR of BCAR3 are utilized and may prevent regulation by tRF5-Glu **(A)** Schematic diagram of the 3’UTR of BCAR3 designating the APA site (black box) and the predicted site for tRF5-Glu binding (grey box). The length of the cloned 3’UTRs for BCAR3 are depicted starting from the stop codon of BCAR3. The primers used for qRT-PCR are designated by solid arrows for the amplicon in the coding region and hatched arrows are used to designate the 3’UTR specific primers. **(B)** Relative expression of the longest BCAR3 3’UTR to total BCAR3 mRNA assayed by qRT-PCR for PEO4, PEO1, OVCAR3, 2008 and SKOV3 cells. qRT-PCR experiments are replicates of three independent experiments and error bars refer to +/- standard error.

The discrepancy between the regulation of BCAR3 by mimics of tRF5-Glu in some ovarian cancer cell lines in contrast to that of SKOV3 and 2008 cells raised the possibility that the 3’UTR of BCAR3 might have been truncated due to the use of an alternative tandem APA in the UTR and 5’ to the tRF5-Glu binding site. A poly-A site prediction search was conducted to determine if a tandem poly-A site is present in the BCAR3 3’UTR [43] (Figure 6A). A qRT-PCR assay relating the total BCAR3 mRNA expression to the longer 3’UTR was developed and substantiated the hypothesis that a shorter UTR was used in SKOV3 and 2008 cells, while the more distal poly-A site was frequently utilized in PEO1, PEO4 and OVCAR3 cells (Figure 6B). Further confirmation was achieved by amplifying and cloning the BCAR3 3’UTR from the poly-A containing fraction of total RNA from PEO4 and 2008 cell lines (Supplementary Table 2).

The qRT-PCR analysis and cloning of the 3’UTR sequences in PEO4 cells confirmed that in this cell line the distal poly-A is more frequently used, while in 2008 proximal sites may be used for poly-A addition. The use of the proximal poly-A site closest to the stop codon in BCAR3 prevents regulation of BCAR3 by tRF5-Glu because the binding site is not present in the shortened BCAR3 mRNA. This finding implies that the regulation of BCAR3 is more complicated than previously known and may vary between cell lines.

### Over expression of tRF5-Glu reduces ovarian cancer cell proliferation

**Figure 7:**
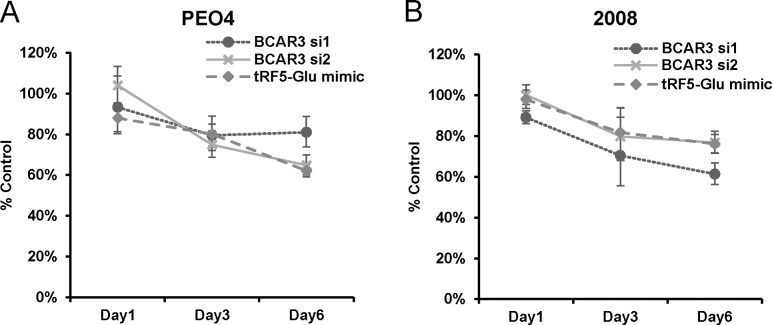
Proliferation of ovarian cancer cells is inhibited by either siRNA to BCAR3 or mimics of tRF5-Glu **(A)** PEO4 cells transfected with either a control siRNA, siRNAs targeting BCAR3 or mimics of tRF5-Glu were grown in culture for up to six days and then fixed and assessed by SRB assay. Proliferation measures the overall cell number resulting from the balance of cell division and cell death over time. Values are shown as percent of control (control siRNA transfected cells; set to 100%) at day one, day three and day six. **(B)** 2008 cells were transfected and grown as described above for PEO4 cells. Cell proliferation experiments are replicates of three independent experiments and error bars refer to +/- standard error. p-value was less than 0.05 for PEO4 and 2008 cells treated with siRNAs to BCAR3 and tRF5-Glu mimic on day six.

It had previously been shown that BCAR3 expression increased proliferation of breast cancer cells [29]. We hypothesized that knocking down BCAR3 expression in ovarian cancer cells would similarly inhibit the proliferation (that is, overall cell number resulting from the balance of cell death and cell division) of ovarian cancer cells. BCAR3 expression was down regulated with siRNAs targeting BCAR3. In both PEO4 and 2008 cells siRNAs targeting the coding region of BCAR3 were able to block BCAR3 expression (Supplementary Figure 2). The loss of BCAR3 and subsequent reduction of proliferation had not previously been shown in ovarian cancer cells. In Figure 7 we show that siRNA inhibition of BCAR3 results in a significant decrease of proliferation when compared to control.

Additionally, studies of tRFs in cancer cells have previously shown that increasing tRF concentration may decrease cancer cell proliferation. For example the over expression of tRF-1001 (tRF3-Ser) has been shown to decrease proliferation of prostate cancer cells [13]. Thus, it was logical to assess the ability of tRF5-Glu to alter the proliferation rate of ovarian cancer cells in culture. We found that mimics of tRF5-Glu significantly reduced the proliferation of both PEO4 and 2008 cells (Figure 7). These results indicate that BCAR3 is not the only target of tRF5-Glu because mimics of tRF5-Glu do not alter BCAR3 expression in 2008 cells (Figure 5A). Therefore, tRF5-Glu would be expected to bind to additional mRNA target sites in additional genes, similar to the mechanism of canonical microRNAs where multiple targets are common. Future studies will be needed to identify additional targets of tRF5-Glu.

## DISCUSSION

Ovarian cancer is known to have the highest mortality rate of the gynecologic malignancies [46]. The high mortality rate is often attributed to late stage of diagnosis, lack of treatment options and the molecular heterogeneity of the disease [46, 47]. Next-generation sequencing is becoming routine in the clinical setting and adds to our understanding of tumor heterogeneity, however, much work is needed to define useful information from the massive amounts of data now clinically available [48]. The noncoding RNAs, including the microRNAs are frequently found extensively expressed in next-generation sequencing studies and are adding to our understanding of tumor heterogeneity [4]. Even more recently many ribosomal RNA and tRNA fragments have been described and the tRFs are now being implicated in cancer biology [49].

Studies of a variety of tRFs have shown that they are actively generated from mature tRNAs and the 5’tRFs are frequently generated by cleavage from tRNAs by ANG. ANG is a well characterized RNase previously associated with the process of angiogenesis, one of the hallmarks of cancer [50]. The biogenesis of tRF5-Glu has previously been shown to involve cleavage by ANG in response to respiratory syncytial virus [51]. We applied similar methods to confirm that ANG is responsible for the biogenesis of tRF5-Glu in ovarian cancer cell lines. siRNA inhibition of ANG followed by northern analysis and ligation PCR confirmed that with reduced expression of ANG, there is reduced expression of tRF5-Glu.

tRFs have been confirmed to function in the stress response to regulate stress granule formation [52] as well as to directly target the RNA binding protein, YBX1, and prevent binding to its mRNA targets [53]. Two other tRFs are known to directly bind mRNA targets in a manner similar to canonical microRNAs [22, 25]. Further indication that tRFs may function to regulate RNA targets has come from their inclusion in AGO-CLIP data sets [12, 23, 54]. The CLASH data set is particularly informative because of its ability to link RNA-RNA hybrids, and indicates that both tRF5-Glu and a variant of tRF5-Glu, homologous to the former miR-2476, are able to target mRNAs in an AGO1 dependent manner [23]. As tRFs are further characterized it is expected that they will contribute to the complexity of tumor heterogeneity.

BCAR3 was especially interesting as a potential target of tRF5-Glu because it has been extensively studied in breast cancer as a protein associated with anti-estrogen resistance [55]. BCAR3 has not been studied in ovarian cancer and little is known regarding its regulation at the mRNA level. Thus, we chose to examine this protein for its potential as a target of tRF5-Glu with the expectation that it would lead to new understanding of a regulatory mechanism previously undefined in this disease. Modifying the method of miR-Catch and using two luciferase reporter assays we were able to confirm that tRF5-Glu binds directly to the BCAR3 mRNA. The BCAR3 specific probe allowed the pulldown of the BCAR3 mRNA and the associated tRF5-Glu and was not pulled down with a random probe. Luciferase assays confirmed the predicted site in the 3’ region of BCAR3 was capable of binding mimics of tRF5-Glu and allowing down regulation of the target. We were also able to confirm that tRF5-Glu is capable of targeting BCAR3 mRNA expression in multiple cell lines, while in still others it appeared incapable of regulating BCAR3 protein expression. APA sites have been shown to alter microRNA regulation in rapidly growing cells and provide another layer of regulation through the 3’UTR of mRNA [44]. Our finding that BCAR3 has an APA site upstream from the tRF5-Glu binding site suggests that this may provide an alternative mechanism for the regulation of BCAR3.

The expression of BCAR3 in breast cancer cells has been previously associated with increased proliferation [29]. In contrast, tRF-1001 has been reported to inhibit prostate cancer cell proliferation [13]. Taken together these two findings led to our hypothesis that increased expression of tRF5-Glu would reduce the proliferation of PEO4 cells, due to direct binding of tRF5-Glu in the 3’ UTR of BCAR3. However, our studies also indicate that mimics of tRF5-Glu reduced the proliferation of 2008 cells, suggesting that other targets of tRF5-Glu are regulated in the 2008 cell line. These results and the regulation of apolipoprotein E receptor 2 (APOER2) by tRF5-Glu demonstrate that, much like microRNA regulators, tRF5-Glu has multiple targets [25].

Future studies will be required to identify the impact of tRF5-Glu expression and regulation of its targets in ovarian cancer and its impact for patient outcome. The study of tRFs is just beginning and this group of noncoding RNAs may open the way for the development of new therapeutic targets as more information is gathered. tRFs add another layer of complexity to discerning the heterogeneity of gene expression and regulatory mechanisms of noncoding RNA.

## MATERIALS AND METHODS

### Cell culture and reagents

Ovarian cancer cell lines PEO1, PEO4, SKOV3, 2008, OVCAR3 were provided by Dr. Monique Spillman [34–36] and HEK293T cells were obtained from ATCC (American Type Culture Collection, Manassas, VA). All the cell lines were grown in RPMI 1640 with 10% fetal bovine serum and Plasmocin (Invivogen, San Diego, CA). Estrogen depletion and addition studies were conducted using cells cultured in Dulbecco's modified Eagle's medium (DMEM) supplemented with 5% charcoal stripped fetal bovine serum [56]. Cells were incubated at 37°C with 5% CO_2_ and have been tested to be mycoplasma free.

### RNA extraction, qRT-PCR and cloning of PCR products

Total RNA was extracted from cells using miRNeasy (Qiagen, Valencia, CA) as previously described [57]. Equal amounts of RNA were reverse transcribed into cDNA using the miScript II RT Kit and qRT-PCR was conducted with miScript SYBR Green (Qiagen). mRNA expression was normalized to beta actin, while small RNA expression was normalized to RNU6B (RNU6B_13, Qiagen). The polyA site was identified in BCAR3 from PEO4 and 2008 cells using Thermoscript RT (Invitrogen) and oligo dT as previously described [58]. PCR products were amplified using the 5’ forward BCAR3 primer with the Universal reverse primer from Qiagen and amplified using GoTaq Green (Promega, Madison, WI). Primers are listed in Supplementary Table 3. PCR products were cloned into T-vector (Life Technologies, Carlsbad, CA) and sent for direct sequencing (University of Minnesota Genomic Center, Minneapolis, MN).

### Quantification of tRFs by ligation qRT-PCR

The sequence of the adapters, probes and primers for tRF quantification by Taqman qRT-PCR are included in Supplementary Table 3. The ligated tRFs were prepared with methods described by Honda et al., [21]. RNA extracted from siRNA treated cells was treated with T4 polynucleotide kinase (T4PNK) to convert the 3’ cyclic phosphate group into a hydroxyl group prior to ligation of a 3’ linker and then copied into cDNA. A forward primer specific to either tRF5-Glu or U6 and a reverse primer specific to the linker were used to amplify targets from the total cDNA pool. Ligated RNA were subjected to qRT-PCR using the QuantiTect Probe RT-PCR Kit (Qiagen). The quantified tRF5-Glu levels were normalized to RNU6B.

The probe placement was determined by Sanger sequencing of the PCR product which identifies the base pairs at the junction of the tRF5-Glu and the linker (Supplementary Figure 3). The sequence identified the specific variant of tRF5-Glu to be one base longer than that previously predicted to be cleaved by ANG [37]. Once the junction between the linker and the most 3’ base of tRF5-Glu and of U6 were determined we were able to design probes for ligation PCR (Supplementary Table 3).

### Northern blot

Northern blot analysis was modified from methods previously described [59]. Denaturing polyacrylamide gels (15%) containing 7M urea was used to separate small RNA. Equivalent loading and RNA integrity were confirmed by gel imaging of the ribosomal RNA using a Licor FC imager. Blots were probed with the 5’-biotinylated RNA probe: tRF5-Glu probe (Supplementary Table 3), and the microRNA marker miR -21 probe was used for size analysis (New England Biolabs, Ipswich, MA).

### Western blot

Protein was extracted from cells using Passive Lysis Buffer (Promega) and samples were loaded onto 4%-20% Mini-PROTEAN TGX gel (Bio-Rad Laboratories, Hercules, CA). The primary antibodies include rabbit anti-BCAR3 #A301-671A (Bethyl Laboratories, Montgomery, TX), ANG 1 (C-20): sc-1408 (Santa Cruz Biotechnology, Dallas, TX), rabbit anti-β-actin #4970 (Cell Signaling Technologies, Danvers, MA). All westerns were repeated two or more times with similar results for each cell line and representative westerns with more than one cell line are included at full size in the Supplementary Figure 4.

### Transfection with siRNA, mimics and inhibitors

Cells were seeded 24h prior to transfection in 35mm petri dishes or 24-well plates and transfected with Lipofectamine 2000 (Invitrogen) according to manufacturer's instructions. The Allstars negative control and the miScript inhibitor negative control were used in the transfection at a concentration of 1 nM (Qiagen). Mimics and inhibitors for tRF5-Glu (MSY0012067 and MIN0012067, Qiagen) were also used in the transfection at a concentration of 1 nM. The siRNAs were obtained from Qiagen and consisted of Gene Solution siRNA product number 1027416 as a control. Two siRNAs were selected from each set of four for further use including Hs_BCAR3_3 SI00053102 (BCAR3 siRNA 1), Hs_BCAR3_6 SI0381603 (BCAR3 siRNA 2), Hs_ANG_6 SI02780197 (ANG siRNA 1) and Hs_ANG_7 SI03071866 (ANG siRNA 7).

### Luciferase assay and constructs

Luciferase constructs containing either 471 base pairs of the BCAR3 3’UTR or a 50 base pair region containing the predicted binding site and a second construct, containing site directed mutations in the predicted binding site were prepared using methods as previously described [60]. The BCAR3 3’-UTR including the predicted binding site for tRF5-Glu was cloned into psiCheck-2 dual luciferase vector. Mutant BCAR3 3’UTR luciferase vectors were generated using primers designed to exchange a C for a G and a G for a C in the tRF5-Glu predicted binding region. These plasmids and a tRF5-Glu mimic were co-transfected into HEK293T cells. Finally, luciferase assays were conducted using the dual luciferase reporter kit (Promega). All primer sequences for vector construction are included in Supplementary Table 3.

### miR-Catch and BCAR3 mRNA pulldown

Five variants of BCAR3 are listed in GenBank and each variant was subjected to unifold (IDTDNA.com) to identify regions that would remain single stranded and available for probe binding. A biotinylated probe targeting the BCAR3 mRNA was designed such that it would bind to a predicted single stranded region of the BCAR3 coding sequence and conjugated to a streptavidin labeled magnetic bead. A similar biotinylated probe was generated from a randomized sequence and used as a negative control. Cell lysates from PEO4 cells following 48 hours of Estrogen stimulation were collected by resuspending and lysing cells in passive lysis buffer (Promega) and hybridizing for 90 minutes at 37°C with either the BCAR3 specific probe or the randomized probe. Magnetic beads were then captured on a Mylteni separator and washed with 1X PBS 3 times. The flow through and magnetic particles were collected for RNA extraction. RNA was converted to cDNA for qRT-PCR amplification and analysis for BCAR3 mRNA and tRF5-Glu were conducted. All primer sequences for miR-Catch probes are included in Supplementary Table 3.

### Sulforhodamine B (SRB) proliferation assay

SRB assays were used to measure cell proliferation, essentially as described by Skehan et al., [61], with a few modifications [62, 63]. The SRB assay measures total cellular protein, which has been shown to be linear with cell number [61]. PEO4 and 2008 cells were seeded in 48 well-plates at a concentration of 1×10^4^ cells/ml and grown under the described experimental conditions for up to 6 days. The cells were then fixed with ice-cold methanol containing 1% acetic acid. Subsequently, cells were incubated with 0.5% SRB solution for 1h at 37 °C, then rinsed 3-5 times with 1% acetic acid washed to remove unbound dye, and then air-dried. Bound SRB was eluted with 10mM Tris, pH10, and the absorbance at 540 nm measured in a multi-well plate reader. Results were expressed as a percentage of signal of the controls.

### Statistical analysis

All the data were expressed as means with standard error and represent at least three independent experiments. Comparison of gene expression was made by using the Student's t-test. A p-value less than 0.05 was considered to be significant.

## SUPPLEMENTARY MATERIALS FIGURES AND TABLES



## Abbreviations

tRFs: tRNA fragments
tRF5-Glu: 5’ fragment of tRNA-Glu-CTC
BCAR3: Breast Cancer Anti-Estrogen 3
ANG: angiogenin
AGO: Argonaute
UTR: untranslated region
RNA-seq: RNA sequencing
qRT-PCR: quantitative Real Time -PCR
APA: alternative poly A site
APOER2: Apolipoprotein E Receptor 2
SRB: Sulforhodamine B.
