# Supplementary material for: A tRNA fragment, tRF5-Glu, regulates BCAR3 expression and proliferation in ovarian cancer cells
Source: Oncotarget. 2017 Sep 8;8(56):95377–91. doi: 10.18632/oncotarget.20709 (PMC5707028; doi:10.18632/oncotarget.20709)
Supplement: Supplementary file 1 [file oncotarget-08-95377-s001.pdf]

A tRNA fragment, tRF5-Glu, regulates BCAR3 expression and proliferation in ovarian cancer cells

SUPPLEMENTARY MATERIALS

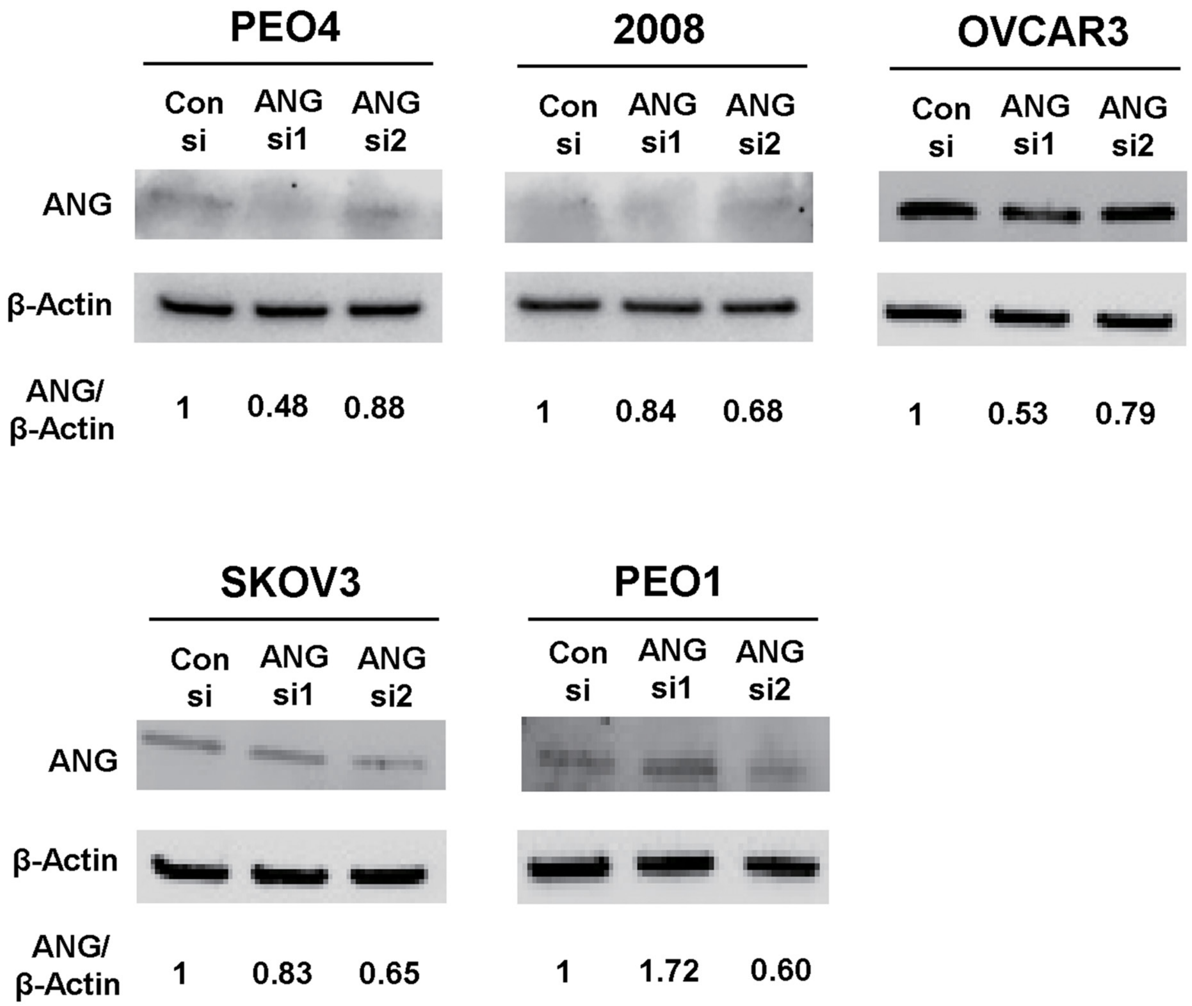

Supplementary Figure 1: Western analysis of ANG protein expression following 48 hours treatment with control siRNA (con si) and ANG siRNAs (ANG si1, 2) in five ovarian cancer cell lines. Densitometry of each band was measured and the relative ratio of ANG/b-Actin is shown.

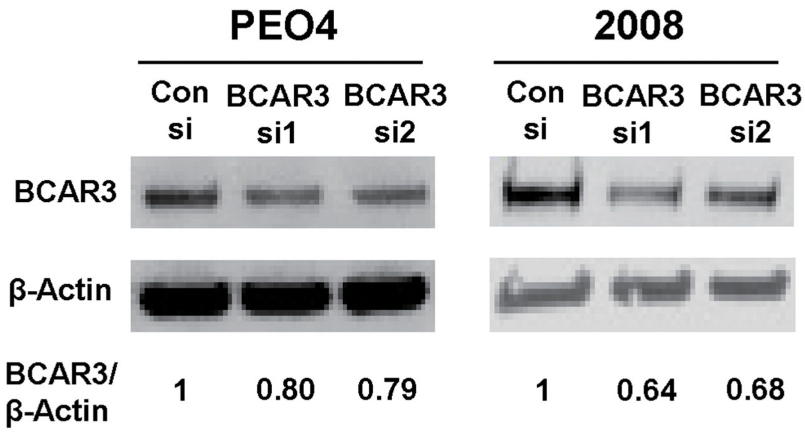

Supplementary Figure 2: Western analysis of BCAR3 protein expression following 48 hours treatment with control siRNA (con si) and BCAR3 siRNAs (BCAR3 si1, 2) in PEO4 and 2008 ovarian cancer cell lines. Densitometry of each band was measured and the relative ratio of BCAR3/b-Actin is shown.

ligated tRF5-Glu

tRF5-Glu forward primer **tRF5-Glu probe**

CCCTGTGGTCTAGTGGTTAGGATTCGGCGCTCTC*GAACACTGCGTTTGCTGGCTTTGAGAGTTCTACAGTCCGACGATC*

3'linker reverse primer

ligated RNU6

U6 forward primer **U6 probe**

GATGACACGCAAATTCGTGAAGCGTTCCATATTTTT*GAACACTGCGTTTGCTGGCTTTGAGAGTTCTACAGTCCGACGATC*

3'linker reverse primer

Supplementary Figure 3: Ligation PCR detects a specific variant of tRF5-Glu or U6. Ligation PCR provides a sensitive method of detecting tRFs and is expected to be more sensitive than Northern analysis. Although multiple cleavage sites are known to be present, Ligation PCR will only detect one variant. Additional variants of either U6 or tRF5-Glu would require additional probes across the junction of the 3' end of the RNA and the 3' adaptor.

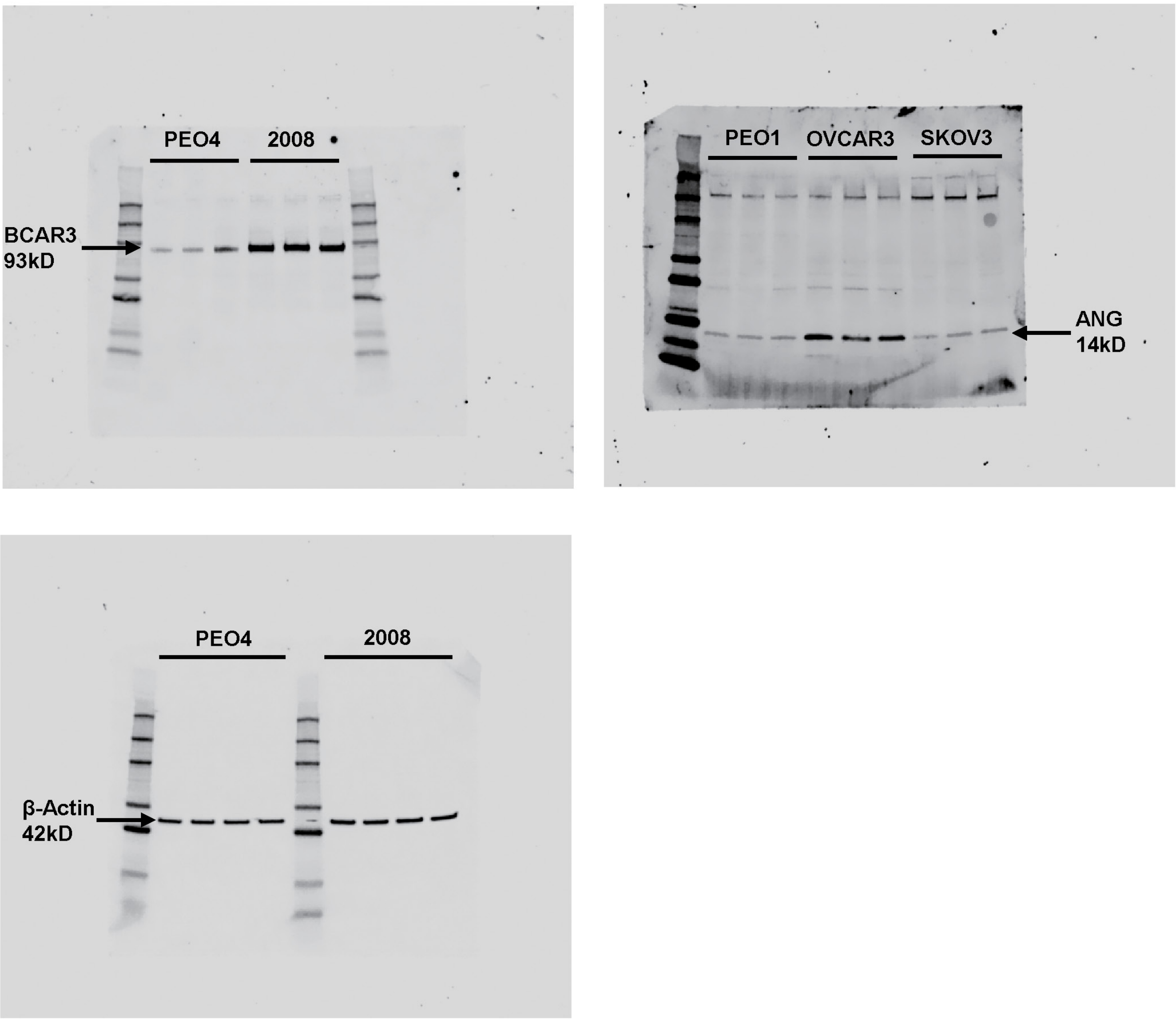

Supplementary Figure 4: Representative Western Blot images of BCAR3, ANG and β-Actin proteins in ovarian cancer cell lines.

Supplementary Table 1: Predicted Cancer Related Targets of miR-2476

Targets of mIR-2476 with a previous association with cancer. TargetScan 6.2 (http://www.targetscan.org/vert_61/)	Total Context+ score	Previous studies in cancer for each of the top genes.	Selection for further study
CBX5 (HP1α)	-0.78	Regulated by an epigenetic methylation [1].	No
PCGF2 (Mel-18)	-0.63	The 3' untranslated region was shown to be associated with tumor suppression [2].	No
CUX1 (CUTL1)	-0.52	Not the gene associated with ovarian cancer at 7q22 [3].	No
BCAR3	-0.50	Associated with anti-estrogen resistance in breast cancer [4].	Yes
TCF20 (SPBP)	-0.43	Very little was known about this gene so not studied [5].	No
CNTN2 (Tag-1)	-0.41	Early studies mostly in neuronal tumors [6].	No
HIF3A	-0.41	Not as much reported about HIF3A as about HIF1 or HIF2 could be interesting in future studies [7].	No
FLT1	-0.41	This is becoming significant in ovarian cancer but at the time these studies were initiated it was not as well studied [8].	No

REFERENCES

1. Thomsen R, Christensen DB, Rosborg S, Linnet TE, Blechingberg J, Nielsen AL. Analysis of HP1α regulation in human breast cancer cells. Molecular carcinogenesis. 2011; 50: 601-13.
2. Ishiwatari H, Nakanishi K, Kondoh G, Hayasaka N, Li Q, Yamashita A, Inoue H, Hakura A. Suppression of tumor growth by the 3′ untranslated region of mel-18 in 3Y1 cells transformed by the E6 and E7 genes of human papillomavirus type 18. Cancer letters. 1997; 117: 57-65.
3. Neville P, Thomas N, Campbell I. Loss of heterozygosity at 7q22 and mutation analysis of the CDP gene in human epithelial ovarian tumors. International journal of cancer. 2001; 91: 345-9.
4. van Agthoven T, Sieuwerts AM, Meijer-van Gelder ME, Look MP, Smid M, Veldscholte J, Sleijfer S, Foekens JA, Dorssers LC. Relevance of breast cancer antiestrogen resistance genes in human breast cancer progression and tamoxifen resistance. Journal of Clinical Oncology. 2009; 27: 542-9.
5. Gburcik V, Bot N, Maggiolini M, Picard D. SPBP is a phosphoserine-specific repressor of estrogen receptor α. Molecular and cellular biology. 2005; 25: 3421-30.
6. Rickman DS, Tyagi R, Zhu X-X, Bobek MP, Song S, Blaivas M, Misek DE, Israel MA, Kurnit DM, Ross DA. The gene for the axonal cell adhesion molecule TAX-1 is amplified and aberrantly expressed in malignant gliomas. Cancer research. 2001; 61: 2162-8.
7. Greer SN, Metcalf JL, Wang Y, Ohh M. The updated biology of hypoxia-inducible factor. The EMBO journal. 2012; 31: 2448-60.
8. Wimberger P, Chebouti I, Kasimir-Bauer S, Lachmann R, Kuhlisch E, Kimmig R, Süleyman E, Kuhlmann JD. Explorative investigation of vascular endothelial growth factor receptor expression in primary ovarian cancer and its clinical relevance. Gynecologic oncology. 2014; 133: 467-72.

Supplementary Table 2: 3'UTR sequences of BCAR3 from Ovarian Cancer Cell Lines

Representative sequences of the 3' UTR of BCAR3

**Cell line**		**Sequence**
PEO4	full length	CCTGTAAAGCAGGCAGAGCTTTGATAACTCTCCAGAGAACCT<br>TTAGAATATCTTTTCAAGTTTCCCCAGCTTCATCTTTGGGAAA<br>GCTTACTGTTTTTGATAAAGTAATAATGTGCAAATCTGACAA<br>TATACAAGCTTTTAGTATCCACAGGATATTAAACGTGTAAATT<br>GCACAGAGCACACTTATTTATGAATTGTCTAAAGTTACTAC<br>TGATTTTAAAATGAATAATTTATTATTAAGGTAACTACTGCTA<br>ATGTTGATCAGCAAATTTAAGAGAAGACCTAGCTATGTTGGC<br>TGGTTGCTTTCTATTATCATGGTATTTGACCATTTTAGTTTTAA<br>TTCCATGTCAGATAAGTGTAAATAGAAGAGTTTAAAAGCA<br>TGAAACATTTCAGAAGGTATCAGTTATATGATATTCTTTAA<br>ACAAATATGAAAAATGTAAATACTCAT<br>GAATGAAAATACATCTTTTTGTG
2008	full length	CCTGTAAAGCAGGCAGAGCTTTGATAACTCTCCAGAGAACCT<br>TTAGAATATCTTTCCAAGTTTCCCCAGCTTCATCTTTGGGAAA<br>GCTTACTGTTTTTGATAAAGTAATAATGTGCAAATCTGACAATA<br>TACAAGCTTTTAGTATCCACAGGATATTAAACGTGTAAATTGCA<br>CAGAGCACACTTATTTATGAATTGTCTAAAGTTACTACTGATTTT<br>AAAATGAATAATTTATTATTAAGGTAACTACTGCTAATGTTGATC<br>AGCAAATTTAAGAGAAGACCTAGCTATGTTGGCTGGTTGCTT<br>TCTATTATCATGGTATTTGACCATTTTAGTTTTAATTCCATGTCAG<br>ATAAGTGTAAATAGAAGAGTTTAAAAGCATGAAACATTTCA<br>GAAGGTATCAGTTATATGATATTCTTTAAACAAATATGAAA<br>AATGTAAATACTCATGAATGAAAATACATCTTTTTGTG
2008	224 bp	CCTGTAAAGCAGGCAGAGCTTTGATAACTCTCCAGAGAACCTT<br>TAGAATATCTTTTCAAGTTTCCCCAGCTTCATCTTTGGGAAAGC<br>TTACTGTTTTTGATAAAGTAATAATGTGCAAATCTGACAATATACA<br>AGCTTTTAGTATCCACAGGATATTAAACGTGTAAATTGCACAGAGC<br>ACACTTATTTATGAATTGTCTAAAGTTACTACTGATTTTAAAATG
2008	125 bp	CCTGTAAAGCAGGCAGAGCTTTGATAACTCTCCAGAGAA<br>CCTTTAGAATATCTTTTCAAGTTTCCCCAGCTTCATCTTTGG<br>GAAAGCTTACTGTTTTTGATAAAGTAATAATGTGCAAATCTGAC

Supplementary Table 3: Primer and Probe List

Primer Type	Sequence and information about the primer design
adj-tRF5-Glu	ggCCCTGTGGTCTAGTGGTTAGGATTC<br>adjusted to achieve improved melting temperature
RNU6B forward primer	Commercially available RNU6B_13. It is critical that RNU6B_13 be used because RNU6B-11 does not work for these assays (Qiagen).
BCAR3 forward	TTGAAAGACACAACACAATGGCCATCGG
BCAR3 reverse	GGAACACATGTGGACTCTCTGCCTTC (cross exon)
Beta actin forward [1]	ATCCACGAAACTACCTTCAACTC
Beta actin reverse[1]	GAGGAGCAATGATCTTGATCTTC (cross exon)
3’RNA adaptor [2]	5’/5Phos/GAACACUGCGUUUGCUGGCUUUGAGAGUUCUACAGUCCGACGAUC/3ddC/-3’
tRF5-Glu probe	5’-/56FAM/CGCTCTCGA/ZEN/ACACTGCGTTTGC/3IABkFQ/-3’
RNU6B probe	5’-/56FAM/TTTGAACAC/ZEN/TGCGTTTGCTGGC/3IABkFQ/-3’
tRF5-Glu forward	CCCTGTGGTCTAGTGGTTAGGATTC
3’ adaptor reverse primer [2]	CGTCGGACTGTAGAACTCTCAAAGC
Luciferase construct BCAR3	The amplified region included 415 of the 458 base pairs reported in GenBank as the 3’UTR of the human BCAR3 (NM_001261408.1).
Xho1 BCAR3	aacctcgagCCTGTAAAGCAGGCAGAGCTTTGA
Not1 BCAR3	aaggatataagcggccgcgAATATCATATAACTGATACCTTCTGA<br>AATGTTTC
BCAR3 mut forward	CAAGCTTTTAGTATCCAGACGATATTAAACGTG
BCAR3 mut reverse	CACGTTTAATATCGTCTGGATACTAAAAGCTTG
BCAR3 3’end forward	AGAGAAGACCTAGCTATGTTGGCTGGTTG
Qiagen Universal reverse	Use Qiagen universal reverse with BCAR3’ end forward and with tRF5-Glu adjusted primers.
Biotin BCAR3 capture probe	5’-/5BiotinTEG/ATGGCCATTGTGTTGTGTCTTTCAATTATG-3’
Biotin random capture probe	5’-/5BiotinTEG/GATTAGTGTCACAACTTTTGTTCGTTGTTG-3’
Xho1 50bp BCAR3	TCGAAGTAATAATGTGCAAATCTGACAATATACAAGCTTTTAGTATCCACAGGATAT
Not1 50bp BCAR3	GGCCATATCCTGTGGATACTAAAAGCTTGTATATTGTCAGATTTGCACATTATTACT
50bp BCAR3 mut forward	TCGAAGTAATAATGTGCAAATCTGACAATATACAAGCTTTTAGTATCCAGACGATAT
50bp BCAR3 mut reverse	GGCCATATCGTCTGGATACTAAAAGCTTGTATATTGTCAGATTTGCACATTATTACT

REFERENCES

1. Sugita M, Haney JL, Gemmill RM, Franklin WA. One-step duplex reverse transcription–polymerase chain reaction for quantitative assessment of RNA degradation. Analytical Biochemistry. 2001; 295: 113-6.
2. Honda S, Loher P, Shigematsu M, Palazzo JP, Suzuki R, Imoto I, Rigoutsos I, Kirino Y. Sex hormone-dependent tRNA halves enhance cell proliferation in breast and prostate cancers. Proceedings of the National Academy of Sciences. 2015; 112: E3816-E25.
